# Ambulatory Neuroproprioceptive Facilitation and Inhibition Physical Therapy Improves Clinical Outcomes in Multiple Sclerosis and Modulates Serum Level of Neuroactive Steroids: A Two-Arm Parallel-Group Exploratory Trial

**DOI:** 10.3390/life10110267

**Published:** 2020-10-31

**Authors:** Gabriela Angelova, Tereza Skodova, Terezie Prokopiusova, Magdalena Markova, Natalia Hruskova, Marie Prochazkova, Marketa Pavlikova, Sarka Spanhelova, Ivana Stetkarova, Marie Bicikova, Lucie Kolatorova, Kamila Rasova

**Affiliations:** 1 Department of Rehabilitation Medicine, Third Faculty of Medicine, Charles University, Ruska 87, 10000 Prague 10, Czech Republic; gabriela@list.sk (G.A.); terezie.tluchorova@gmail.com (T.P.); orgoluza@centrum.cz (M.M.); natalia0087@gmail.com (N.H.); prochazkova777@gmail.com (M.P.); marketa@ucw.cz (M.P.); 2 Department of Steroids and Proteofactors, Institute of Endocrionology, 11694 Prague, Czech Republic; tskodova@endo.cz (T.S.); mbicikova@endo.cz (M.B.); lkolatorova@endo.cz (L.K.); 3 Department of Rehabilitation and Sport Medicine, Motol University Hospital, V Uvalu 84, 150 06 Prague 5, Czech Republic; sspanhelova@seznam.cz; 4 Department of Neurology, Third Faculty of Medicine, Charles University, 10000 Prague, Czech Republic; ivana.stetkarova@fnkv.cz

**Keywords:** multiple sclerosis, physical therapy, neuroproprioceptive facilitation and inhibition, neuroactive steroids, dehydroepiandrosterone, cortisol

## Abstract

Background: Only few studies have monitored the potential of physical activity training and physical therapy to modulate the reaction of the endocrine system. In this study, the effect of neuroproprioceptive facilitation and inhibition physical therapy on clinical outcomes and neuroactive steroids production in people with multiple sclerosis was evaluated. Moreover, we were interested in the factors that influence the treatment effect. Methods: In total, 44 patients with multiple sclerosis were randomly divided into two groups. Each group underwent a different kind of two months ambulatory therapy (Motor program activating therapy and Vojta’s reflex locomotion). During the following two months, participants were asked to continue the autotherapy. Primary (serum level of cortisol, cortisone, 7α-OH-DHEA, 7β-OH-DHEA, 7-oxo-DHEA, DHEA) and secondary (balance, cognition and patient-reported outcomes) outcomes were examined three times (pre, post, and washout assessments). Results: In both groups, there is a decreasing trend of 7-oxo-DHEA concentration in post-assessment and 7β-OH-DHEA in washout versus pre-assessment. A higher impact on neuroactive steroids is visible after Vojta’s reflex locomotion. As for clinical outcomes, the Paced Auditory Serial Addition Test and Multiple Sclerosis Impact Scale significantly improved between post-assessment and washout assessment. The improvement was similar for both treatments. Conclusions: Neuroproprioceptive facilitation and inhibition improved the clinical outcomes and led to non-significant changes in neuroactive steroids. Trial registration (NCT04379193).

## 1. Introduction

Multiple sclerosis is a chronic, inflammatory, demyelinating and neurodegenerative immune-mediated disease of the central nervous system that causes a range of clinical dysfunctions and limits the quality of life and active participation within it [1].

Its etiology is linked to a variety of genetic and environmental factors [2]. Alterations in the endocrine system, for example in hypothalamic–pituitary–adrenal axis function, can contribute to the pathogenesis and progression of this disease [3]. It is also the balance in neuroactive steroids that seems very important in the development of the disease [4,5]. Decreases in dehydroepiandrosterone (DHEA) and its derivatives’ levels were observed in some neurodegenerative neuropsychiatric disorders [6,7,8] including multiple sclerosis [9,10].

DHEA, together with its derivatives (mainly oxidized metabolites or hydroxylated at position 7, namely 7α-hydroxy-DHEA, 7β-hydroxy-DHEA or 7-oxo-DHEA) [11,12,13], is one of the most abundant steroid hormones in the human body. Outside the adrenal cortex it is synthesized in the glia in the brain [8,14] and it acts as a positive modulator of the N-methyl-D-aspartate receptor [15]. DHEA has a wide range of biological functions, and the most important in the pathogenesis of multiple sclerosis may be the following: it reduces inflammatory processes, modulates cellular immunity, has neuroprotective effects [12,16,17,18], improves cognitive functions, strengthens memory, protects against apoptosis and antagonizes effects of oxidizing agents and glucocorticoids [8], plays a role in myelination [19], and partakes in the priming of synaptic plasticity induction [6,8,13,15,20,21,22,23]. In co-operation with other hormones and transmitters, it affects some aspects of human mood, modifies some features of human emotions and behavior and increases feelings of well-being [8].

Even though physical therapy, a part of rehabilitation in multiple sclerosis [1], primarily treats functions and promotes functional independence, prevents complications and enhances the overall quality of life [24], it has a potential to influence the production of neuroactive steroids. There are two ways to modulate their production: by physical activity training [25], and by neuroproprioceptive facilitation and inhibition physical therapy [26].

Physical activity (fitness/endurance/resistance) training acts as an acute stress. Adrenal glands respond by the production of adrenaline (which stimulates the instant stress hormone response), and of cortisol and DHEA (which create a short- and long-term stress hormone response [27,28,29]). Major stress response systems such as the hypothalamic–pituitary–adrenal axis and the autonomic nervous systems, influence immune function (cytokine regulation) and thus may influence the disease activity [30,31], and minimize demyelination through the stimulation of Th2 shift [32]. Until now, only two studies have explored the potential effect of physical activity training on the endocrine system in multiple sclerosis [33,34]. While Heesen et al. 2003 [33] confirmed that aerobic training significantly increases adrenalin, noradrenalin and adrenocorticotropic hormones, Schulz et al. [34] did not find any significant changes in these hormones.

Neuroproprioceptive facilitation and inhibition physical therapy addresses the cerebellum through sensorimotor stimuli applied in different postural positions. Due to its connection with the limbic system (via the Papez circuit) [35] and the stimulation of long-term potentiation (the basic mechanism of learning and memory) [24], it has a potential to affect the hypothalamus through the hypothalamic–pituitary–adrenal axis [25], and thus other systems, including the immune and endocrine. This possibility was explored only in our pilot study [36], where we were interested whether the motor program activating therapy influences DHEA level. There we observed slight but statistically non-significant correlations between changes of interleukins and DHEA.

In this study, we hypothesized that two kinds of neuroproprioceptive facilitation and inhibition physical therapy, motor program activating therapy and Vojta’s reflex locomotion, would improve clinical outcomes immediately after the two-month ambulatory program, and that this improvement would persist two months later when participants continued with the auto therapy. We expected that the clinical improvement would be accompanied by the increased levels of DHEA and its derivatives, and decreased levels of cortisol and cortisone. Moreover, we hypothesized that other factors (ender, age, disease duration, Expanded Disability Status Scale, long-term corticosteroid treatment) would influence the effect of the therapy.

## 2. Methods

### 2.1. Study Design

In the Two-Arm Parallel-Group Exploratory Trial (NCT04379193), realized between May 2015 and May 2017, participants underwent two kinds of neuroproprioceptive facilitation and inhibition physical therapy (motor program activating therapy and Vojta’s reflex locomotion). The period of ambulatory, face-to-face therapeutic program took two months (1 h, twice a week). During the following two months, participants were asked to continue with the auto therapy (they learnt it during the therapeutic program), but not to attend any new exercise or therapeutic sessions or make adjustments to their medications.

Primary outcomes (serum level of cortisol, cortisone, 7α-OH-DHEA, 7β-OH-DHEA, 7-oxo-DHEA, DHEA) and secondary outcomes (balance, cognition and patient-reported outcomes) were examined three times (pre, post, and washout assessments).

### 2.2. Participants

Participants were recruited from the database of the Centre for diagnosis and Treatment of Multiple Sclerosis, Kralovske Vinohrady University Hospital in Prague. For inclusion into the study they were selected by a neurologist based on their anamnesis and clinical assessment (not on any serum analysis). Accordingly, participants were both genders, adults, diagnosed with definite multiple sclerosis [37] of moderate to severe disease severity (Expanded Disability Status Scale (EDSS) [38] ranging from 3 to 7.5), and without severe orthopaedic or cardiovascular dysfunction or presence of another neurological disorder. They should be at least 6 months without individual physiotherapy, at least 2 months without relapse and change of pharmacological treatment, and women should not use oral contraceptives (Table 1).

The Fisher exact test was used to compare categorical characteristics between therapy groups. The *t*-test was used to compare continuous characteristics between therapy groups.

All subjects signed an informed consent form approved by the Ethics Committee of Kralovske Vinohrady University Hospital in Prague (full trial protocol EK-VP/22/0/2014 is available there). Chosen participants were randomly divided into two groups in a 1:1 ratio by an independent study coordinator, using computer-generated randomization. The sample size was estimated using the Berg Balance Scale (BBS) change difference as the main outcome endpoint. Based on the previous study [39] with a similar setting and therapy, we estimated the number of participants to be 20 in Group 1 and 20 in Group 2 to have 90% power to detect a difference in BBS ≥3 points at the 5% level of statistical significance.

### 2.3. Interventions

#### 2.3.1. The Therapy

Both groups underwent 16 sessions. Therapists were maximally helpful and adopted a schedule for each patient to complete all the sessions. To increase the adherence, effective reminders during the therapy were used. The treatment in each session was led face-to-face by well-educated (MSc.) experienced therapists (minimally two years’ practice with people with multiple sclerosis) specially trained in each method, and it was modified according to the patient’s status and reaction to the therapy. The physical load during all therapies was fairly light [40], but the demands being induced on the central nervous system were high [41].

Participants in Group 1 underwent motor program activating therapy (MPAT). They were corrected into a position that fits functional ontogenesis where the joints are functionally centered. Then, the somatosensory (manual and verbal) stimuli were applied to activate motor programs in the brain, which then led to the muscle co-contraction of the patient’s whole body when the patient was lying, sitting, standing up or moving forward. Activated programs were repeated under various conditions and in different situations and environments to teach patients to use the acquired motor skills in their daily life automatically [42].

Participants in Group 2 underwent Vojta’s reflex locomotion (VRL), which is based on the activation of the global movement patterns stored in the central nervous system. For their facilitation/activation, the following setups were used: four initial positions (supine, prone, side lying, low kneeling position), angular setting of extremities and stimulation of the activation points (zones), defined by precise localization and pressure direction and their combination (space and time summation). These rich afferent stimuli are processed in the central nervous system, which has a global reflex reaction with not only a motor but also a sensory and autonomic response. There are two global movement patterns (reflex turning and reflex creeping) used for the treatment, which are activated involuntarily. The activation of global movement patterns enriches the patient’s spontaneous movement via automatic control of the body position, upright mechanism and goal-directed isolated movement [43].

#### 2.3.2. The Auto Therapy

During their treatment sessions both groups learnt to use the principals of each method (mainly the correction into the position that fits functional ontogenesis) in the usual activities of daily living. Specifically, they tried to repeat qualitatively correct and coordinated movement patterns in various situations.

### 2.4. Examination

A blinded assessor (unaware of the intervention assigned to the assessed participant) examined the primary and secondary outcomes immediately before the beginning (pre-assessment), immediately after (post-assessment) and 8 weeks after the end of the two months’ physical therapy program (washout assessment).

#### 2.4.1. Primary Outcomes Measures

The blood was collected in the morning between 7 and 8 a.m. (in the follicular phase of the menstrual cycle in women). Samples were collected in plastic tubes, frozen and stored at −79 °C. Levels of cortisol, cortisone, 7α-OH-DHEA, 7β-OH-DHEA, 7-oxo-DHEA and DHEA were quantified by the LC-MS/MS method using triple stage quadrupole–mass spectrometer. It employs 500 μL of human plasma and 3000 μL of CSF extracted with diethyl ether and derivatized with 2-hydrazinopyridine. It had been validated in terms of sensitivity, precision and recovery. For more details, see Sosvorova et al., 2015 [44].

#### 2.4.2. Secondary Outcomes Measures

The clinical measures were focused on balance (Berg Balance Scale (BBS) [45] and Time Up and Go test (TUG) [46]), cognitive functions (Paced Auditory Serial Addition Test (PASAT 3)—single digits were presented every 3s [47]) and patient-reported outcomes (Multiple Sclerosis Impact Scale (MSIS-29) [48], Modified Fatigue Impact Scale (MFIS) [49], Visual Analogue Scale (VAS) for Walking and Balance [31], Multiple Sclerosis Walking Scale (MSWS-12) [50]).

### 2.5. Data and Statistical Analysis

Continuous data were summarized as means with standard deviations (SD) or medians with interquartile range (IQR) wherever appropriate. Categorical variables were summarized using absolute and relative frequencies. The effect of the therapy was tested using a paired *t*-test or paired Wilcoxon test (immediate effect of therapy between the second and the first assessment, long-term effect of therapy between the third and the first assessment). A two-sample *t*-test or Wilcoxon two-sample test (sum rank test) were used to compare continuous variables between therapy groups.

In general, non-parametric techniques (median, Wilcoxon, Kruskall–Wallis) were used with time-related measurements (e.g., TUG test) and serum level of neuroactive steroids, otherwise parametric methods were employed. Because of the many applied tests, there was a higher chance of Type I error, i.e., rejection of the null hypothesis while it is true. To address this issue, *p*-values were adjusted by the Benjamini–Hochberg method for multiple comparisons.

Acknowledging that factors such as gender age, disease duration, EDSS and long-term corticosteroid treatment (prednisone, methylprednisolone) can highly modify both therapy effectiveness and serum levels of neuroactive steroids, we used mixed linear regression models with the measured variable as a response variable, the group and treatment phase as independent variables, and the patients’ characteristics as a confounding factor. Random intercept for each individual allowed for unrecognized variability between patients. The interaction between treatment phase and treatment group, and possible interactions of the group and phase with the characteristics were explored as well. All analyses were performed in statistical language and environment R, v. 5.0.3.

## 3. Results

### 3.1. Baseline Characteristics

**Figure 1:**
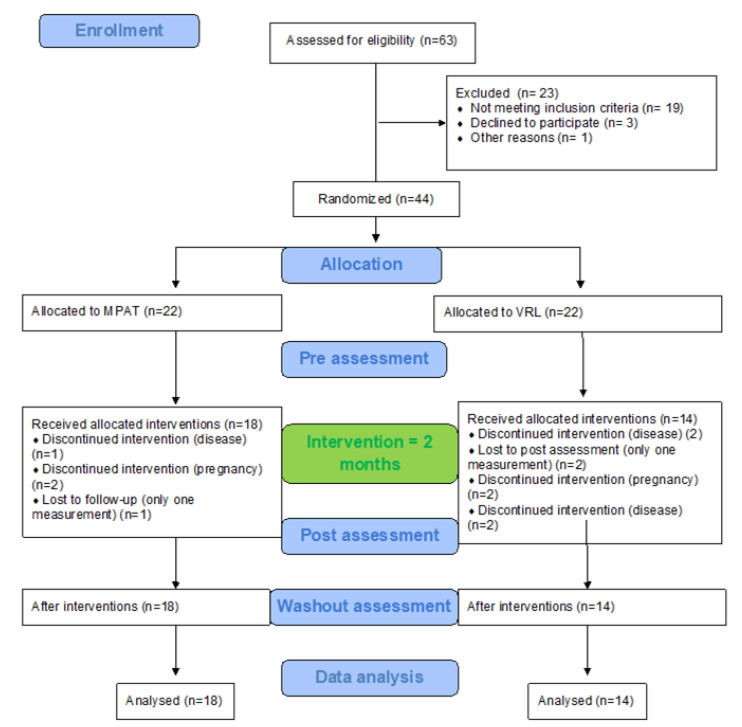
Flow chart diagram.

From 63 eligible people with multiple sclerosis, 44 consented and were randomly divided into two groups (22 into Group 1 and 22 into Group 2). Of these, 18 participants in Group 1 and 14 in Group 2 underwent the whole program (all three measurements) (Figure 1).

Groups significantly differed at baseline characteristics in age (Group 1 was older), type of the disease (in Group 2 secondary progressive type prevailed), its duration (Group 1 had longer disease duration) and sex distribution (in Group 2 there were more males), while the groups were similar in Expanded Disability Status Scale, Body Mass Index, height and weight (Table 1). Groups also significantly differed in some baseline clinical outcomes (Paced Auditory Serial Addition Test was worse in Group 1) and neurohormones (7-oxo-DHEA was significantly higher in Group 2) (Table 2).

In the case of TUG and neurohormones, a nonparametric approach was appropriate. The median was used as a location parameter, the paired Wilcoxon test was used to test for change in a measurement and the two-sample Wilcoxon test was used to compare differences between therapy groups.

For other variables, a parametric approach was appropriate. Mean was used as a location parameter, paired *t*-test was used to test for change in a measurement and two-sample *t*-test was used to compare differences between therapy groups.

### 3.2. The Effect of the Therapy (Irrespective of the Type of Therapy)

Participants significantly improved in cognitive functions measured by PASAT 3 (by mean 2.6 points, *p* = 0.010), which further improved after the next two months (by mean total change 3.9 points, *p* < 0.001), and in the impact of multiple sclerosis evaluated by MSIS-29 (by 4.6 points, *p* = 0.044). This clinical improvement was followed by a decrease in the median (0.054 nmol/L (*p* = 0.022)) of 7-oxo-DHEA.

### 3.3. Differences between Groups

There was a significant difference between the groups in the change of balance measured by the BBS score (while Group 1 improved by 1 point, Group 2 worsened by 1 point, *p* = 0.036) in post-assessment. In particular, motor program activating therapy showed a trend toward the improvement of cognitive functions measured by the PASAT 3 (2.9 points, *p* = 0.07) and balance measured by TUG (−1 s, *p* = 0.034). Vojta’s reflex locomotion showed a trend toward the improvement of cognitive functions measured by the PASAT 3 (2.1 points, *p* = 0.053) and subjective evaluation of walking by VAS (0.9 points, *p* = 0.09) in post-assessment. Moreover, the groups differed in hormonal response to the therapy. Vojta’s reflex locomotion had a higher impact on neuroactive steroids. It led to an immediate significant decrement in cortisone (median −13.0 nmol/L, *p* < 0.001), 7β-OH-DHEA (−0.09 nmol/L, *p* = 0.019) and 7-oxo-DHEA (−0.12 nmol/L, *p* = 0.001), while hardly any change was observed following motor program activating therapy. Differences between groups were statistically significant (cortisone (*p* = 0.0223), 7β-OH-DHEA (*p* = 0.0232) and 7-oxo-DHEA (*p* = 0.0053)) (Table 2).

### 3.4. Influence of Other Factors on the Treatment Effect

**Figure 2:**
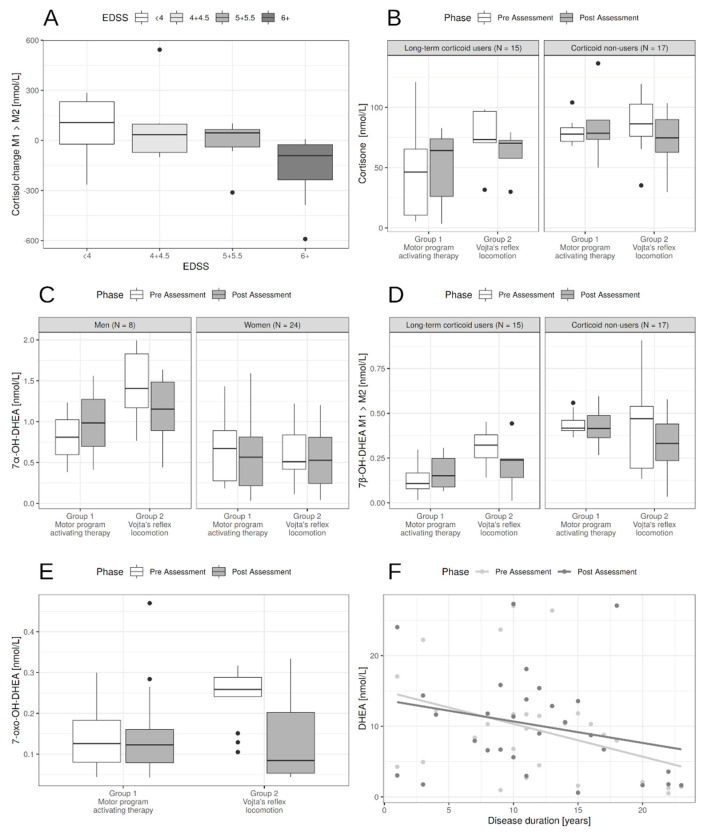
Influence of patients’ characteristics on neurohormone levels. (**A**). The effect of EDSS on cortisol change between post- and pre-assessment. The higher EDSS the higher decrease in cortisol levels. (**B**). Long-term corticoid users had lower levels of cortisone, mostly in the motor program activating therapy (MPAT group). For patients with Vojta’s reflex locomotion PT (physical therapy) lowered the levels compared to the MPAT group. (**C**). Men had higher 7α-OH-DHEA levels pre-assessment and sex had no significant effect on change during PT. (**D**). Long-term corticoid users had lower levels of 7β-OH-DHEA. For patients with Vojta’s reflex locomotion PT (physical therapy) lowered the levels compared to the MPAT group, irrespectively of corticoid use. (**E**). Effect of Vojta’s reflex locomotion PT compared to MPAT on 7-oxo-DHEA. There was also a baseline effect of EDSS—the higher the EDSS the lower the 7-oxo-DHEA (not shown). (**F**). While PT did not influence DHEA levels, the effect of disease duration was pronounced.

Univariately, the baseline levels of some neuroactive steroids were significantly influenced by sex (7α-OH-DHEA (*p* = 0.00) were significantly lower for women compared to men)) and long-term corticosteroid treatment (significantly lower level of cortisol (*p* = 0.054), cortisone (*p* = 0.007), 7α-OH-DHEA (*p* = 0.023)7β-OH-DHEA (*p* < 0.001) and DHEA (*p* = 0.004) compared to those not treated by corticosteroids). While long-term corticosteroid treatment influences the baseline level of neuroactive steroids, it does not significantly influence the effectiveness of physical therapy (Table 3 and Figure 2).

Of other patient-related factors, the following decreased baseline levels of some neurohormones: type of multiple sclerosis in cortisol (*p* = 0.015) and cortisone (*p* = 0.024), disease duration in DHEA (*p* = 0.017), and the level of disability measured by EDSS in 7α-OH-DHEA (*p* = 0.033) and DHEA (*p* = 0.023). Apart from EDSS influence on cortisol levels, there was no statistically significant influence of these factors on the effect of PT (Table 3 and Figure 2).

For cortisol, the higher EDSS, the slightly lower the baseline cortisol levels, but more importantly the higher the decrease in cortisol levels in post-assessment (*p* = 0.014, Figure 2A). There was also a slight effect of age, but both age and MS type effect disappeared with the inclusion of EDSS because of the correlation between these factors (Table 3).

## 4. Discussion

Exploring whether and how PT influences neuroactive steroids is innovative and could help to develop a novel approach enabling the regulation of the complex neuro-endocrine–immune crosstalk.

Until now, only a few studies have investigated the level of neuroactive steroids in connection with exercise or physical therapy [27,51,52]. We were the first to look for their response in multiple sclerosis, extending the exploration of our pilot project [36], wherein a trend for the correlation between changes of interleukins and DHEA had been documented. In the present study, the design was improved by increasing the amount of participants (although the amount was still limited due to the long duration of the study), by monitoring the effect of two kinds of neuroproprioceptive facilitation and inhibition physical therapy (motor program activating therapy and Vojta’s reflex locomotion), by monitoring not only DHEA but also cortisol and cortisone, and finally by looking for immediate (post-assessment) and persistent (washout assessment) effect. On the other hand, there were several limitations. The exact evaluation of neuroactive steroids in serum was difficult due to the adaptive processes of cortex glandulae suprarenali. It would be better to find them in cerebrospinal fluid [53], or in the whole steroid metabolome of adrenal origin, which could reflect neuroactive steroids’ changes even in serum. To understand this topic fully, serum concentrations of other neuroactive mediators and markers of multiple sclerosis, such as serum neurofilament light, tumor necrosis factor α, interleukin 1β, neutralizing antibodies against interferon beta, and the analysis of oligoclonal bands and other markers in cerebrospinal fluid, could have been monitored. However, this was beyond the scope of the present study, and should be considered in the future research. The next limitation was the non-uniform distribution of participants within groups, which could be caused by the drop-out after randomization. Although all participants fulfilled inclusion criteria and were divided into groups independently, they differed at some baseline characteristics, clinical outcomes, and the level of neurohormones. On the other hand, this study offers information about personalized rehabilitation in ‘real-life’ settings.

Changes in neuroactive steroids in connection with exercise or physical therapy were monitored in multiple sclerosis in our pilot study [36], in the elderly [27], in thyroidectomized women [51], and in postmenopausal females [52]. Heaney et al. [27] described that DHEA levels significantly increased and cortisol significantly decreased immediately after acute exercise in the elderly. They did not find a difference between PT intensity in two groups, the first of which did moderate activities (golf, yoga, badminton, swimming and keep fit classes) and the second did endurance training (running, and cycling, circuit training and karate). Jandova et al. [51] found a significant decrease in the unconjugated DHEA and a significant increase in cortisol after the health resort treatment including balneotherapy and PT in thyroidectomized women. Honcu et al. [52] described the activation of adrenal steroidogenesis (an increase in DHEA, 7α-OH-DHEA, but cortisol too) together with the improvement of mood balance in postmenopausal females after spa treatment based on physical activity. In the present study, a trend towards the reduction in 7-oxo-DHEA in post-assessment and 7β-OH-DHEA in washout versus pre-assessment was documented, similarly as in Heaney et al. [27], but in contrary with Jandova et al. [51] and Honcu et al. [52]. The studies are inconsistent in studied populations or interventions, so the mechanism by which neurohormones could be influenced still remains unclear.

The significant changes of neuroactive steroids in this study were mainly driven by Vojta’s reflex locomotion. While cortisone decreased significantly as we expected, 7β-OH-DHEA and 7-oxo-DHEA significantly decreased in spite of our expectations. Our expectations were based on the literature, where glucocorticoids (cortisol and cortisone) are widely described as immunosuppressive mediators [54], with a generally catabolic effect that increases with age [28]. Their effects should be balanced out by DHEA, which has the opposite effect—activating the immune system, building up tissues and acting as an anti-aging factor [55].

Our next expectation, that the neurohormones’ changes following motor program activating therapy would be significant, was not confirmed. It was probably caused by a lesser effect on clinical outcomes in comparison with our previous research, wherein a significant effect on muscle strength, spasticity [42], skill motor, walking [42,56], fatigue, depression and quality of life [56] was documented. In this study, only a trend towards the improvement of PASAT 3 (in contrast to our previous study [42], while in accordance with our other study [56]) and TUG (similar to our previous study [39]), and a significant difference between motor program activating therapy and Vojta’s reflex locomotion in BBS (in accordance with previous studies [42,56]), was found. Bias could be caused by the heterogeneity of people with multiple sclerosis between groups. Although all participants fulfilled the inclusion criteria and were divided into groups independently, they differed in baseline characteristics (except for EDSS and anthropometric parameters), including the level of neuroactive steroids. On the other hand, this study brings information about personalized rehabilitation in ‘real-life’ settings (participants were recruited by neurologists from the Centre for diagnosis and Treatment of Multiple Sclerosis according to inclusion criteria, but also according to the motivation to participate in a four-month study with such an invasive intervention as blood collection).

Furthermore, our next hypothesis concerning long-term therapeutic effect was not confirmed. Based on our previous findings, whereby the improvements of muscle strength, spasticity, skill motor, walking [42] and cognitive functions [42] were documented two months after finishing the ambulatory program, we were convinced that physical therapy could start the adaptive and plastic processes throughout the human body, and therefore it would prevent the progression of multiple sclerosis. Unfortunately, we did not find any long-lasting effect in this study, similarly as in other studies [57,58]. Only indirect findings indicate the importance of long-term treatment. Our participants significantly worsened on the Multiple Sclerosis Impact Scale without motor program activating therapy.

Regressive analysis brought out interesting points about the role of sex and long-term corticosteroid therapy. Sex influences the disease progression and inflammatory activity of multiple sclerosis [59]. Our findings confirmed its impact on the level of neuroactive steroids (7α-OH-DHEA and 7-oxo-DHEA were significantly lower in women at baseline level). Sex hormones could negatively influence clinical outcomes, which significantly worsen stability in the ovulation phase [60]. In this study, we examined patients strictly in the follicular phase. The dependence of PT impact on sex was expected but not confirmed (except for 7α-OH-DHEA). From our research, it follows that in future studies it would be better to analyze male and female patients separately. Long-term corticosteroid treatment significantly decreases the baseline levels of cortisol, cortisone, 7α-OH-DHEA and 7β-OH-DHEA; however, it has no significant impact on PT effect. On the other hand, a chronically decremental endocrine system is still able to react to physical therapy.

## 5. Conclusions

Neuroproprioceptive facilitation and inhibition physical therapy improve clinical outcomes and modulates the serum level of neuroactive steroids.

Motor program activating therapy and Vojta’s reflex locomotion significantly differ in their effects on neuroactive steroids and clinical outcomes (in cortisone, 7β-OH-DHEA and 7-oxo-DHEA and Berg Balance Scale). After Vojta’s reflex locomotion cortisone, 7β-OH-DHEA and 7-oxo-DHEA decreased significantly, while after the motor program activating therapy there were only non-significant changes (DHEA increases). Neuroactive steroid changes do not persist for more than two months without physical therapy.

A better understanding of the underlying mechanisms of neuroactive steroids as the complex neuro-endocrine–immune crosstalk targeted by physical therapy is an important issue for people with multiple sclerosis and needs further clinical studies.

## Figures and Tables

**Table 1:** Baseline characteristics of participants.

Participants Characteristic	All Groups (N = 32)	Motor Program Activating Therapy (N = 18)	Vojta’s Reflex Locomotion (N = 14)	F-Test *p*-Value	*t*-Test *p*-Value
Female/Male (%)	22/10 (68%/32%)	16/2 (89%/11%)	6/8 (43%/57%)	**0.0964**	
Age (years)—mean (SD; range)	46.9 (12.9; 19–71)	51.9 (11.6; 32–71)	40.6 (11.9; 19–63)		**0.0110**
Type of multiple sclerosis (counts: CIS; RR; SP; PP)	1; 20; 10; 1	0; 9; 9; 0	1; 11; 1; 1	**0.0152**	
Disease duration (years) -mean (SD; range)	12.4 (7.4; 1–38)	15.4 (7.8; 4–38)	8.5 (4.9; 1–15)		**0.0066**
EDSS—mean (SD; range)	4.4 (1.7; 1–7.5)	4.6 (1.5; 1–7.5)	4.2 (1.9; 1–6.5)		0.5712
Corticosteorid treatment (counts and%: yes/no)	15/17 (47%/53%)	10/8 (56%/44%)	5/9 (36%/64%)	0.307	
BMI (kg m^−2^)—mean (SD; range)	24.2 (4.3; 16.4–36.2)	24.5 (4.8; 16.4–36.2)	23.9 (3.7; 18.1–31.7)		0.6906

Note: BMI = Body Mass Index, EDSS = Expanded Disability Status Scale. Types of multiple sclerosis (MS): CIS = clinically isolated syndrome, RR = relapsing–remitting, SP = secondary–progressive, PP = primary–progressive. SD = standard deviation, **bold** = statistically significant difference.

**Table 2:** Immediate and persistent treatment effects of neuroproprioceptive facilitation and inhibition physical therapy of motor program activating therapy (Group 1) and Vojta’s reflex locomotion (Group 2).

	Group 1 + 2					Group 1					Group 2					Difference in Immediate Therapeutic Effect between Group 1 and 2	Difference in Persistent Therapeutic Effect between Group 1 and 2
Part A: Variables Where Non-Parametric Approach Appropriate	M1	M1 → M2	Wilcoxon Test	M1 → M3	Wilcoxon Test	M1	M1 → M2	Wilcoxon Test	M1 → M3	Wilcoxon Test	M1	M1 → M2	Wilcoxon Test	M1 → M3	Wilcoxon Test		
	Median	Median Change	*p*-Value	Median Change	*p*-Value	Median	Median Change	*p*-Value	Median Change	*p*-Value	Median	Median Change	*p*-Value	Median Change	*p*-Value	W-Test	W-Test
TUG (s)	9.6	−0.6	0.106	−0.3	0.35	11.3	−1	0.034	−0.6	0.56	9.1	−0.1	0.889	−0.3	0.507	0.106	0.927
Cortisol (nmol/L)	384	−12	0.681	−36	0.19	363	17	0.766	−39	0.42	399	−39	0.235	−27	0.250	0.323	0.792
Cortisone (nmol/L)	74.6	−5.5	0.190	−0.4	0.99	68.53	4.2	0.580	5.8	0.3	85.7	−13.0	0.000 *	−5.3	0.078	**0.022**	**0.047**
7a-OH-DHEA (nmol/L)	0.74	−0.03	0.595	0.04	0.7	0.67	0.04	0.459	−0.03	0.82	0.90	−0.18	0.173	0.06	0.313	0.128	0.773
7b-OH-DHEA (nmol/L)	0.36	−0.03	0.119	−0.06	**0.04**	0.3	0.01	0.821	−0.07	0.08	0.43	−0.09	**0.017**	−0.06	0.297	**0.045**	0.751
7-oxo-DHEA (nmol/L)	0.18	−0.05	0.022	−0.03	0.19	0.13	0.00	0.890	0.03	0.5	0.26	−0.12	0.003 *	−0.09	**0.014**	**0.002**	**0.024**
DHEA (nmol/L)	8.58	0.21	0.808	−0.87	0.33	8.37	0.80	0.495	−1.18	0.63	9.03	−0.43	0.715	−0.95	0.250	0.448	0.897
**Part B: Variables Where Parametric Approach Appropriate**	**M1**	**M1 → M2**	***t*-Test**	**M1 → M3**	***t*-Test**	**M1**	**M1 → M2**	***t*-Test**	**M1 → M3**	***t*-Test**	**M1**	**M1 → M2**	***t*-Test**	**M1 → M3**	***t*-Test**	**Difference in Therapeutic Effect between Group 1 and 2**	**Difference in Therapeutic Effect between Group 1 and 2**
	**Mean**	**Mean Change**	***p*-Value**	**Mean Change**	***p*-Value**	**Mean**	**Mean Change**	***p*-Value**	**Mean Change**	***p*-Value**	**Mean**	**Mean Change**	***p*-Value**	**Mean Change**	***p*-Value**	***t*-Test**	***t*-Test**
PASAT 3	41.9	2.6	0.010 *	3.9	0.000 *	37.1	2.9	0.07	4.3	**0.01**	47.8	2.1	0.053	3.5	**0.04**	0.681	0.716
BBS	45.4	0.1	0.807	−0.7	0.345	42.5	1.0	0.14	−0.4	0.75	49.2	−1.0	0.127	−1.1	0.15	0.036	0.644
MSIS	66.3	−4.6	**0.044**	0.3	0.923	67.6	−5.3	0.12	−0.4	0.92	64.7	−3.6	0.222	1.2	0.75	0.716	0.785
MFIS	34.8	−2.2	0.236	−1.0	0.601	36.4	−1.8	0.51	−1.9	0.54	32.7	−2.8	0.281	0.2	0.94	0.804	0.590
VAS walking	5.4	0.5	0.295	0.1	0.715	5.3	0.1	0.87	−0.1	0.85	5.5	0.9	0.090	0.5	0.17	0.365	0.445
VAS balance	5.3	0.6	0.176	0.3	0.432	5.0	0.9	0.27	0.2	0.81	5.7	0.4	0.444	0.5	0.32	0.597	0.652
MSWS-29	32.9	1.3	0.441	1.1	0.481	34.2	0.5	0.82	1.3	0.63	31.1	2.3	0.392	0.9	0.53	0.598	0.906

Note: **bold** significant at 0.05, without correction, * significant adjusted *p*-value.

**Table 3:** Factors influencing the treatment effect on neuroactive steroids: mixed linear regression results.

	Phase Post vs. Baseline	Therapy Group VRL vs. MPAT	Interaction Phase * Therapy	Sex Women vs. Men	Corticoid Use	Type of MS PP + SP vs. RR + CIS	Disease Duration	EDSS	Age	Model with Interactions and Individual Factors Together
Cortisol (nmol/L)	0.74	0.68	x	x	0.054	0.015	x	0.014 *	0.047	In presence of Phase * EDSS interaction other terms become non-significant
Cortisone (nmol/L)	0.41	0.055	0.058	x	0.007	0.024	x	0.071	x	Type of MS becomes non-significant
7α-OH-DHEA (nmol/L)	0.61	0.075	0.070	0.005	0.023	x	x	x	x	Both gender and corticoid use remain significant
7β-OH-DHEA (nmol/L)	0.73	0.016	0.019	x	< 0.001	x	x	0.062	x	Both corticoid use and EDSS are significant
7-oxo-DHEA (nmol/L)	0.70	0.003	0.007	x	x	x	x	0.031	x	
DHEA (nmol/L)	0.38	0.43	x	x	0.004	x	0.020	0.023	x	Disease duration becomes non-significant

Note: The table denotes *t*-test *p*-values for the regression terms. All examined mixed linear regression models included the phase, group and interaction between phase and group terms, and one of the patients’ characteristics: sex, long-term corticoid use, type of MS, disease duration, EDSS, age and BMI (not shown as not significant in any model). Selected variables were then further examined in the model together with possible interactions between phase and factor and group and factor—interesting results are mentioned in the last column. * EDSS significant in interaction with phase; while cortisol level baseline is higher for people with higher EDSS, it decreased more by post-assessment. Phase = timing of measurements. MPAT = motor program activating therapy (Group 1), VRL = Vojta’s reflex locomotion (Group 2). Types of multiple sclerosis (MS): CIS = clinically isolated syndrome, RR = relapsing–remitting, SP = secondary–progressive, PP = primary–progressive, EDSS = Expanded Disability Status Scale.

## Abbreviations

TUG—Timed Up and Go Test, BBS—Berg Balance Scale, MSWS-12—Multiple Sclerosis Walking Scale-12, MSIS-29—Multiple Sclerosis Impact Scale, VAS—Visual analogue scale, MFIS—Modified fatigue Impact Scale, M1 Pre-assessment (baseline clinical characteristic), M2 post-assessment, examination 2 (immediate effect), M3 washout assessment, examination 3 (persistent effect), M1 → M2: difference M2-M1, M1 → M3: difference M3-M1.
